# Morphological and molecular characterization of a sexually reproducing colony of the booklouse *Liposcelis bostrychophila* (Psocodea: Liposcelididae) found in Arizona

**DOI:** 10.1038/srep10429

**Published:** 2015-05-27

**Authors:** Qianqian Yang, Zuzana Kučerová, Steve J. Perlman, George P. Opit, Edward L. Mockford, Adi Behar, Wyatt E. Robinson, Václav Stejskal, Zhihong Li, Renfu Shao

**Affiliations:** 1 Department of Entomology, College of Agriculture and Biotechnology, China Agricultural University, Beijing 100193, China; 2 Crop Research Institute, Drnovská 507, 161 06 Prague 6, Czech Republic; 3 Department of Biology, University of Victoria, Victoria, British Columbia V8P 5C2, Canada; 4 Department of Entomology and Plant Pathology, Oklahoma State University, OK 74078, USA; 5 Department of Biological Sciences, Illinois State University, Normal, Illinois 61790/4120, USA; 6 Kimron Veterinary Institute, P.O. Box 12, Bet-Dagan 50250, Israel; 7 GeneCology Research Centre, Faculty of Science, Health, Education and Engineering, University of the Sunshine Coast, Maroochydore DC, Queensland 4556, Australia

## Abstract

The booklouse, *Liposcelis bostrychophila*, is a worldwide pest of stored products. For decades, only thelytokous parthenogenetic reproduction was documented in *L. bostrychophila*. Male *L. bostrychophila* were first found in Hawaii in 2002. In 2009, a sexual strain was found in Arizona. We examined the morphology of both males and females of the Arizona strain and compared the Arizona sexual strain with the Hawaii sexual strain and the parthenogenetic strains of *L. bostrychophila*. The sexual and parthenogenetic strains show some differences in eye morphology. To examine the relationship between sexual and asexual lineages, we sequenced the mitochondrial *12S* and *16S* ribosomal RNA genes of males and females from the Arizona strain. Phylogenetic analyses of *L. bostrychophila* individuals revealed that: 1) the sexually reproducing colony found in Arizona contains two closely related mitochondrial DNA haplotypes – one present in only females and the other in both males and females; and 2) the Arizona sexual strain was most closely related to a parthenogenetic strain in Illinois. We detected *Rickettsia* in all of the parthenogenetic individuals we checked but not in any Arizona sexual individuals. Further evidence is required to establish whether the presence of *Rickettsia* is linked to asexual reproduction in *Liposcelis*.

The booklouse genus, *Liposcelis* Motschulsky (Psocodea: Liposcelididae), contains 126 species^1^. Approximately 10% of the *Liposcelis* species have a close affiliation with human habitation^2^. These wingless, tiny insects (~1 mm body size) occur widely in grain storage facilities, grain processing facilities, and human dwellings where they infest various types of stored products^3^,^4^.

*Liposcelis bostrychophila* (Badonnel) is probably the most widely distributed species in the genus. It has long been known that *L. bostrychophila* reproduces via thelytokous parthenogenesis, i.e. the species has only female individuals^5^,^6^. Parthenogenetic species have the potential to rapidly establish and extend the species range in new locations. This type of asexual reproduction is not rare in Psocoptera and occurs in at least 13 of the 32 families of the three suborders of Psocoptera^7^. Asexual reproduction is considered evolutionarily disadvantageous, owing to the limited opportunities it presents for generation of genetic variation^8^ or to purge deleterious mutations. However, based on allozyme polymorphism and PCR-RFLP studies, considerable variation has been found among populations of parthenogenetic *L. bostrychophila*^8^,^9^. Interestingly, all parthenogenetic *L*. *bostrychophila* investigated harbor a strain of the bacterial endosymbiont *Rickettsia*^10^,^11^,^12^. A number of maternally transmitted bacterial endosymbionts of arthropods, including *Wolbachia*^13^, *Cardinium*^14^ and *Rickettsia*^15^, induce parthenogenesis in their hosts; this strategy is adaptive as it increases the frequency of the transmitting sex (i.e. females). It has thus been suggested that the strain of *Rickettsia* that infects *L*. *bostrychophila* causes parthenogenesis, although this has not yet been conclusively demonstrated.

The discovery of males of *L. bostrychophila* in the Hawaii Archipelago provided the first ever evidence of sexual reproduction in this species^16^. In 2009, two of our authors (E.L.M and A.B., unpublished data) found a sexually reproducing colony of *L. bostrychophila* in Arizona, USA, and succeeded in establishing a lab colony. This colony consists of males and females (Lb_M and Lb_F hereafter), and subsequent observations showed that females were obligatory sexual and needed to be fertilized by males in order to produce offspring, and that both male and female offspring are produced17^17^.

In this study, we used morphological and molecular approaches to examine the relationship between sexual and asexual (parthenogenetic) strains of *L. bostrychophila*. We compared morphological characters in detail among Arizona and Hawaii sexual individuals and parthenogenetic *L. bostrychophila* individuals using optical microscopy (OM) and scanning electron microscopy (SEM). We sequenced portions of the mitochondrial *12S* and *16S* ribosomal RNA genes of 15 booklice from the Arizona colony and 18 booklice from the asexual strains collected in Asia, Africa, America, Australia and Europe, and performed phylogenetic analyses of these and other previously available sequences. Finally, we ask whether there is a link between *Rickettsia* infection and parthenogenesis in *L. bostrychophila*. We screened male and female individuals of the Arizona colony and nine parthenogenetic strains of *L. bostrychophila* for *Rickettsia.*

## Materials and Methods

### Sample collection

Sexual *L*. *bostrychophila* individuals were collected by Edward L. Mockford and Adi Behar, a postdoc in S. Perlman’s lab at the time, in Cochise County, Coronado National Forest, Vista Point on Cave Creek Road, Arizona (elevation 1553 m., N31, 53.105′, W109, 10.403′) in September 2009. Specimens sifted from ground litter in oak forest and collected by aspirator were placed in glass tubes, 95 mm × 19 mm (outside diameter), which had been provided with pieces of oak leaf and other debris from the ground litter to ca. 2 cm deep in the tubes. Several pieces of “Cheerios”™ were added to each tube as primary nutrient source. The tubes were stoppered with cotton and placed in a closed glass container (battery jar) over a saturated KCl solution to keep a high relative humidity and maintained at room temperature (22–24 °C) with an outside opaque cover to reduce light from above. Every few days (initially every day), the tubes were examined under a dissecting microscope to determine if there was mortality and if more food was needed. Additional pieces of Cheerio were added ca. every three weeks, when the pieces in the tubes were seen to be full of holes. In general, the booklice thrived under these conditions, and in ca. 5 months the original single tube of sexual *L*. *bostrychophila* had to be subcultured due to high population density. Males of *L*. *bostrychophila* were always in low numbers but were always present, and an occasional copulation was observed. The cultures have since been maintained in the laboratories at Oklahoma State University and University of Victoria.

The parthenogenetic strains of *L*. *bostrychophila* were collected at 20 locations in 9 countries from grain storage and food-product warehouses, except the Vietnam (Lb_VN) and Seychelles (Lb_SC) samples, which were from imported plant products and a passenger’s belongings inspected by the China Entry-Exit Inspection and Quarantine Bureau in Guangxi and Beijing, respectively. Samples of *Liposcelis corrodens* (Heymons), a closely related species to *L. bostrychophila*, were collected in Central Bohemia (Czech Republic) and Kansas (USA).

Populations of all strains were reared on a wheat flour-based diet and maintained at 27 °C and relative humidity of 75% in 24 h of darkness^18^. Samples of all strains were stored in 75–100% ethanol at –20 °C or –80 °C. Voucher specimens were morphologically identified using diagnostic keys of adults and kept at China Agricultural University (Beijing, China), Oklahoma State University (Stillwater, USA), Crop Research Institute (Prague, Czech Republic) and University of the Sunshine Coast (Australia).

### Microscopic examination

An optical microscope (OM) (PZO, Warsaw) was used initially for the morphological examination of specimens from both the Arizona sexual and parthenogenetic *L. bostrychophila* strains. The taxonomic identification of the species and nomenclature used was as previously described^4^,^16^,^19^ (Table 1). Head width (W) measurements were taken using an optical microscope equipped with an objective micrometer (Table 2).

**Figure 1:**
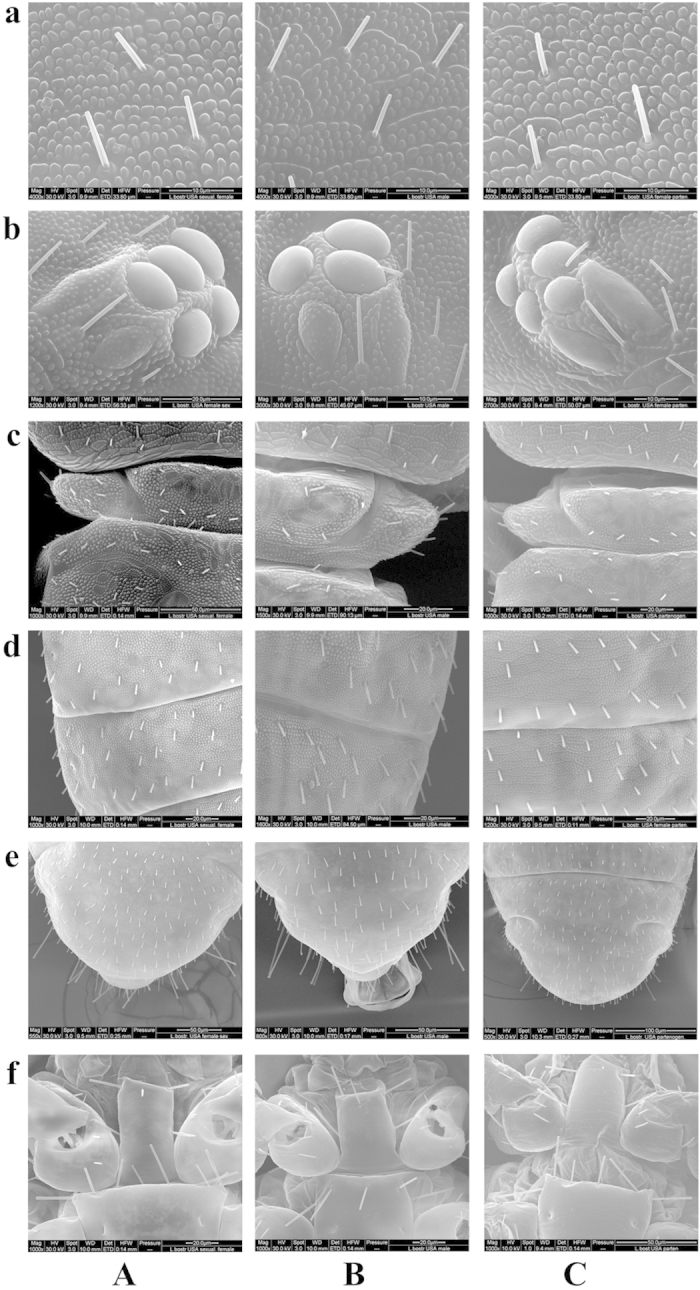
The scanning electron microscope micrographs of *Liposcelis bostrychophila*. Morphological comparison of the vertex sculpture and setae (**a**) compound eye (**b**) lateral lobe of pronotum (**c**) abdominal tergite of 4-5th segment, dorsal view (**d**) terminal abdominal segments, dorsal view (**e**) prosternum and sythoracic sternum setae, ventral view (**f**) in Arizona sexual female (**A**) Arizona sexual male (**B**) Kansas parthenogenetic female (**C**).

Detailed morphological characteristics were studied (Table 1) and illustrated (Fig. 1) using a scanning electron microscope (SEM). The SEM specimens were prepared as described^20^ and subsequently examined using Quanta 200F (FEI, Brno, Czech). The morphological characteristics and surface sculptures were studied at magnifications of 100–20,000×. The origins of strains and numbers of specimens used were summarized in Table 1.

### DNA extraction, amplification and sequencing

Genomic DNA was extracted from individual specimens with CTAB method^21^ or DNeasy Blood and Tissue Kit (QIAGEN), or PrepMan Ultra (Life Technologies Corporation) following the protocol^22^. Fragments of *12S* and *16S* of *L. bostrychophila* and *L. corrodens* were amplified with primer pairs 12SF-12SR^23^ and 16Sar-16Sbr^24^. Amplification was performed in 25 μL final reaction volume containing 0.125 μL TaKaRa Ex *Taq* (5 U/μL), 2.5 μL 10 × Ex *Taq* Buffer, 2 μL dNTP mixture (2.5 mM each), 1 μL of each primer (10 μM) and 1 μL of genomic DNA. PCR cycling conditions for amplification were: 94 °C for 3 min, followed by 35 cycles of 98 °C for 10 sec, 45–50 °C for 30 sec, and 72 °C for 40–90 sec depending on the amplicon size, and finally 72 °C for 8 min. Amplicons were checked by agarose gel electrophoresis.

Purified PCR products were sequenced at Beijing AoKe Biotechnology, Australian Genome Research Facility, or Macrogen USA. Each PCR product was sequenced from both ends with the forward and reverse primers used in PCR amplification. Sequenced fragments were checked in Chromas 1.0^25^ or Geneious^26^ and through BLAST search in NCBI website (http://www.ncbi.nlm.nih.gov/); overlapping sequence fragments were assembled using DNAMAN 5.0 (Lynnon Biosoft). Sequences determined in this study were deposited in GenBank (Table 3). Sequences of *12S* and *16S* of *L. bostrychophila* available from previous studies were retrieved from GenBank and included in phylogenetic analyses conducted in the current study.

### Sequence alignment and phylogenetic analyses

*12S* and *16S* sequences of *L. bostrychophila* and *L. corrodens* were aligned; each gene dataset was analyzed separately (Table 3). Multiple sequence alignments were generated with ClustalW, implemented in MEGA 5.0, using the default options. Pairwise genetic distances were estimated using the Kimura-2-Parameter (K2P) distance model in MEGA 5.0. Phylogenetic reconstruction of *12S* and *16S* sequences, using Bayesian and maximum likelihood methods were performed, with MrBayes v. 3.1.2 and RaxML 7.0.4, respectively.

For Bayesian analyses, two independent runs with four simultaneous Markov chains (three heated and one cold chain) were run for 5 × 10^6^ generations and were sampled every 1,000 generations (average standard deviation of split frequencies <0.01). Majority-rule consensus trees were estimated combining results from duplicated analyses, with the first 25% generations discarded. For the Maximum Likelihood analyses, the recommended models were determined with jModelTest 2.1 for each dataset according to the Bayesian Information Criterion (BIC), using the BEST tree topology search operation and a BioNJ starting tree from five random trees, with 100 bootstrap replicates. Distance analyses were performed using the K2P distance model with 100 bootstrap replicates. Trees were visualized with FigTree.

### Screening parthenogenetic and sexual strains of ***L. bostrychophila*** for ***Rickettsia***. 

Adults of nine parthenogenetic strains and the Arizona sexual strain of *L. bostrychophila* were screened for the presence of *Rickettsia*, using PCR with specific primers (Table 4). We screened five individuals each of the Guangxi (Lb_GX), Beijing (Lb_BJ) and Kansas (Lb_KS) parthenogenetic strains, and one individual each of the Zhengzhou (Lb_ZZ), Croatia (Lb_HR), Xinshagang (Lb_XSG), Sanya (Lb_SY), Vietnam (Lb_VN) and Germany (Lb) parthenogenetic strains. We also screened five males and eight females of the Arizona sexual strain. Each sample was surface-sterilized with 20% commercial bleach solution for 10 min, and rinsed twice with distilled water before DNA extraction^32^.

## Results

### Morphological characteristics of the Arizona sexual strain of ***L. bostrychophila***

Decisive diagnostic features of female specimens of the Arizona sexual strain of *L. bostrychophila* were consistent with those described previously for females of *L. bostrychophila* (Section II, Group D)^4^,^19^. Details of morphological characters and the head width measurements of female and male adult individuals of the Arizona sexual strain of *L. bostrychophila* were summarized in Tables 1 and 2. For sexual females, the body length is 1.09-1.12 mm and the head width is 279.6 ± 7.3 μm; for sexual males, the body length is 0.70-0.75 mm and the head width is 202.1 ± 4.2 μm (Tables 1 and 2). Males were smaller than females, as is the case in other sexual *Liposcelis* species^4^,^19^. The coloration of both females and males are homogeneously chocolate to bistre, with heads slightly reddish-brown and the middle part of anterior margin of tergites darker. The compound eyes of females consist of seven ommatidia with two oval ones with granulated surfaces and five round ones with smooth surfaces, whereas those of males consist of five ommatidia with two oval ones with granulated surfaces and three round ones with smooth surfaces. Tubercles on the vertex of the head are very distinct and of medium size, and they are smaller than the alveoli of small fine hairs. Spindle-shaped areas are well defined and separated by lines of tubercles; the average hair distance is approximately twice their length. One pair of lateral prosternal setae is present on the posterior half of the prosternum, in addition to the setae on the anterior half. The number of mesosternal setae is 6-9 with 8 as the most common in females and 5-6 in males. The SI (humeral setae of pronotum) is short and pointed, and is not much longer than other small fine hairs of the lateral lobe. Abdominal terga 1-2 are divided in two transverse bands each; 3-7 are with distinct tubercles of medium size, smaller than alveoli of small fine hairs. Each presents a pale posterior membranous band with sculpture different from that on the anterior portion of tergum.

Notable morphological differences were found in the Arizona sexual females in the surface structure of eyes and in the number of some thoracic hairs and setae. In Arizona sexual males, differences were found in external eye morphology and phallosome. Comparison of morphological characteristics between the Arizona sexual strain and parthenogenetic strains and between the Arizona sexual strain and the Hawaii sexual strain of *L. bostrychophila* were shown in Table 1 and Fig.1, and described further below.

### Morphological differences between females of the sexual Arizona strain and parthenogenetic strains of ***L. bostrychophila***

Females of the Arizona sexual strain differed from the parthenogenetic strains by having unusually developed surface structure in their compound eyes. All females of the strains for comparison, both sexual and parthenogenetic, had seven ommatidia (Table 1). Arizona sexual females had two oval ommatidia (posterodorsal and posteroventral) on each eye and these ommatidia had granulated surfaces; the other five round ommatidia had normal smooth surfaces (Fig. 1Ab). In parthenogenetic females, all of the seven ommatidia (two oval, five round) had normal smooth surfaces (Fig. 1Cb). Slight differences were also found between the sexual and the parthenogenetic females in: 1) the number of small fine hairs on the lateral lobe of the pronotum, and 2) the number of metasternal setae. Sexual females had more hairs (6-10) than the parthenogenetic ones (3-6). Sexual females had six to nine metasternal setae whereas the parthenogenetic females had five to seven metasternal setae. However, the larger number of hairs may be related to the larger size of the sexual females and should not be viewed as a valid difference.

### Morphological differences between the Arizona sexual strain and the Hawaii sexual strain of ***L. bostrychophila***

The Arizona sexual strain of *L. bostrychophila* differs slightly from the Hawaii sexual strain in eye structure. All males of the Arizona strain (n = 44) had five ommatidia with no variation in the number of ommatidia (Table 1). Two of the ommatidia in each eye were oval and had granulated surfaces (posterodorsal and posteroventral), similar to ommaditia in the eyes of sexual females; the other three ommatidia were round and had normal smooth surfaces (Fig. 1Bb). Unlike in males of the Arizona sexual strain, all of the five ommatidia in males of the Hawaii sexual strain had normal smooth surfaces (one specimen from original ethanol sample^16^). Besides, Arizona males had a pair of basal rods of the phallosome that were not fused at their anterior end (Fig. 1B), i.e. the rods were separate but were so close that they touched or almost touched each other and the space between them was not as wide as in the case of rods in Hawaii males^16^. However, this can result from differences in pressure on the cover slip and should not be viewed as a valid difference. The parameres in Arizona males bear a small denticle on the outer surface near the tip just like in Hawaii males^16^. Contrary to Arizona females, all seven ommatidia of Hawaii females (n = 14) had smooth surfaces. The other decisive diagnostic characters did not differ between the Arizona and Hawaii sexual strains.

### The Arizona sexual strain is closely related to an Illinois parthenogenetic strain of ***L. bostrychophila***

**Figure 2:**
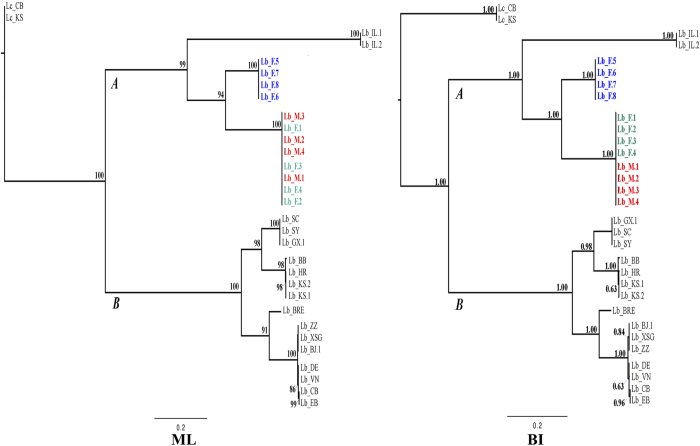
MrBayes inference (BI) and Maximum likehood (ML) phylogenetic trees inferred from partitioning*12S* and *16S* rRNA. Only minor variation in the placement of certain taxa with trees inferred by individual *12S* or *16S* rRNA genes, therefore, only the results derived by partitioning dataset are shown.

**Figure 3:**
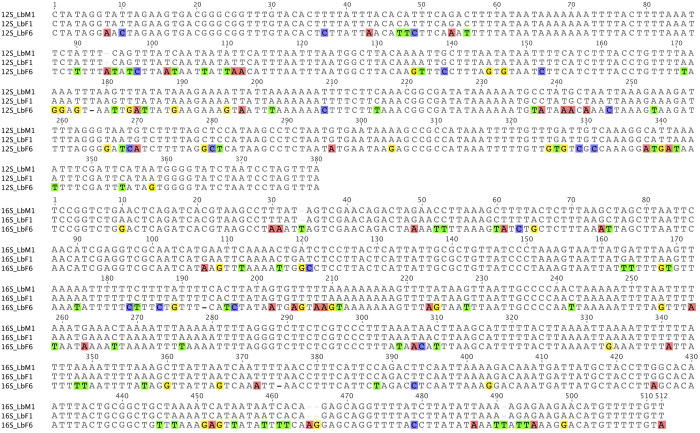
Alignment of *12S* and *16S* sequences from two haplotypes of both females and males of the Arizona strain of *L. bostrychophila*. Each alignment is with three sequences: Lb_M1, Lb_F1 and Lb_F6.

Phylogenetic trees constructed with Bayesian and maximum likelihood methods had similar topologies (Fig. 2). The 38 booklice in our phylogenetic analyses were divided into two major clades with strong support (posterior probability 1; bootstrap value 100%) regardless of the tree-building methods used. *L. bostrychophila* booklice from the sexually reproducing colony in Arizona formed a clade with a parthenogenetic strain from Illinois^28^,^29^,^30^; this clade was well supported (posterior probability 1; bootstrap value 99%). The individuals from the sexually reproducing colony in Arizona were grouped together with strong support and were divided into two groups: all males (Lb_M1-5) and five females (Lb_F1-5) were in one group, whereas the other five females (Lb_F6-10) were in another group. These two groups differ by 18.4% in their *12S* sequences and by 16.6% in their *16S* sequences (Fig. 3). As the 15 individuals in these two groups were from the same colony, it indicates that the sexually reproducing colony of *L. bostrychophila* in Arizona contains two distinct mt DNA haplotypes.

Excluding the Illinois parthenogenetic strain, all other parthenogenetic strains formed another clade (posterior probability 1; bootstrap value 100%). The parthenogenetic strains in this clade were divided into two well-supported groups. One group contained six parthenogenetic strains from Kansas (Lb_KS.1-2), Croatia (Lb_HR), Beibei of China (Lb_BB), Guangxi of China (Lb_GX), Sanya (Lb_SY) and Seychelles (Lb_SC). The other group contained 12 parthenogenetic strains from Brisbane, Australia (Lb_BRE), Winnipeg (Lb_WP), Manhattan, USA (Lb_MH), Australia (Lb_AU), the United Kingdom (Lb_UK), Zhengzhou, China (Lb_ZZ), Xinshagang, China (Lb_XSG), Beijing, China (Lb_BJ), Germany (Lb_DE), Vietnam (Lb_VN), East Bohemia, Czech Republic (Lb_EB) and Central Bohemia, Czech Republic (Lb_CB).

### ***Rickettsia*** is present in parthenogenetic ***L. bostrychophila*** but not in the Arizona sexual strain

*Rickettsia* was detected in all individuals from the parthenogenetic strains of *L. bostrychophila* that we screened (Lb_BJ, Lb_GX, Lb_KS, 5 individuals each; Lb_ZZ, Lb_HR, Lb_XSG, Lb_SY, Lb_VN and Lb_DE, one individual each). We did not detect *Rickettsia* in any individuals from the Arizona sexual strain (n = 13).

## Discussion

The discovery of sexually reproducing *L. bostrychophila* in Arizona confirmed that both sexual and parthenogenetic reproduction modes exist in this species. Species with both sexual and parthenogenetic reproduction modes have been reported previously in other insect orders such as Hemiptera and Hymenoptera^33^,^34^. Thelytokous parthenogenesis is not rare in Psocoptera (barklice+booklice) and occurs in 13 of the 32 families from all of the three suborders^7^,^35^. Some barklouse species or species complexes in Psocoptera have also been shown to contain bisexual, obligate or facultative parthenogenetic reproduction modes^36^,^37^. However, sexual forms of the booklice, *L. bostrychophila*, have only been discovered recently^16^; the current study is the first that investigated comprehensively both the molecular and morphological characteristics of a sexually reproducing *L. bostrychophila* colony.

The Arizona sexual strain of *L. bostrychophila* is very similar to the parthenogenetic strains in the key diagnostic morphological characteristics for the identification of this species^4^. The only notable difference is in eye morphology: the Arizona sexual strain has two oval ommatidia with granulated surfaces whereas the parthenogenetic strains have smooth surfaces in all ommatidia, which is typical for species of the genus *Liposcelis*. Similar eye morphology, i.e. having ommatidia with granulated surfaces, was also shown recently in one strain of the bisexual species *Liposcelis silvarum* (Kolbe)^2^. Both females and males of this *L. silvarum* strain had one oval ommatidium with a granulated surface (slightly different type of granulation than described here in Arizona sexual *L. bostrychophila* strain) in each of their compound eyes whereas the other ommatidia were smooth. Other *L. silvarum* strains examined had ommatidia with normal smooth surfaces^2^. It is important to point out that this difference found in one strain of *L. silvarum* was not sufficient to establish it as a new species^2^. The other slight differences between the sexual and asexual *L. bostrychophila* strains studied were in the number of hairs on the lateral lobe of the pronotum. The body size of individual booklice should not be used as a decisive diagnostic character because of the usually high intraspecific variability of this character in the genus *Liposcelis*^4^. The morphological differences between parthenogenetic and sexual *L. bostrychophila* females that have been described are, therefore, not sufficient to warrant splitting sexual and asexual strains into two species at this time, although they are clearly reproductively isolated by virtue of their mode of reproduction, and a recent study refers to the Arizona sexual strain as L. nr. bostrychophila^17^. As stated previously, sexual *L. bostrychophila* individuals were also collected in Hawaii^16^ with the key difference between the Hawaii and the Arizona strains also in eye structure. Hawaiian individuals have smooth ommatidia, whereas some (two ovals) of the ommatidia in the Arizona individuals have granulated surfaces.

Our molecular phylogenetic analyses showed that the Arizona sexual strain of *L*. *bostrychophila* was closely related to a parthenogenetic strain in Illinois. A recent phylogeographic study of asexual and sexual strains of the psocopteran, *Echmepteryx hageni*, noted high mitochondrial DNA diversity in asexual strains relative to sexual strains^38^. Parthenogenetic reproduction in *L. bostrychophila* is well documented^5^. A number of studies have showed abundant genetic variation among the parthenogenetic strains of *L. bostrychophila*^6^,^8^, suggesting that parthenogenesis has persisted for a long time. However, we refrain from speculation at this time on the evolutionary history and origins of sexual and asexual reproduction in *L*. *bostrychophila*, for a number of reasons. First, the present study only looked at mitochondrial genes, which can show different evolutionary patterns from nuclear genes. Second, little is known about the genetic diversity of sexual strains or wild asexual strains of *L*. *bostrychophila*. Only two sexual strains of *L*. *bostrychophila*, i.e. the Hawaii and the Arizona strains, have been found to date, and human-associated parthenogenetic strains of *L*. *bostrychophila* tend to be sampled more than the wild strains. Third, it is important to experimentally determine whether there is any cryptic or facultative parthenogenesis and/or sexual reproduction in a strain before making any conclusions about reproductive mode. Finally, as far as we are aware, there are no convincing documented examples of re-evolution of sexual reproduction from lineages where it had been previously lost, and it is difficult to rule out alternative hypotheses^39^.

We found two distinct mitochondrial haplotypes in the same colony of the Arizona sexual *L*. *bostrychophila*, with one found only in females, and the other in both males and females. The persistence of distinct mitochondrial DNA haplotypes within insect species may be due to their association with maternally transmitted endosymbionts, including *Wolbachia*^40^,^41^ and *Rickettsia*^42^, although in some cases endosymbionts do not appear to be involved^43^. Interestingly, a recent study of the Arizona sexual population found that mitochondrial polymorphism was associated with extreme sex ratio distortion^17^.

It is intriguing that we did not find *Rickettsia* symbionts in the Arizona sexual strain, as all previously sampled parthenogenetic *L*. *bostrychophila* individuals harbor a strain of *Rickettsia felis*^10^,^12^,^44^; no other maternally transmitted symbionts have been identified and confirmed by sequencing in *L*. *bostrychophila*^10^. Furthermore, we detected *Rickettsia* in all of the parthenogenetic individuals in this study. It has been suggested that *Rickettsia* may cause parthenogenesis in *L*. *bostrychophila*^12^, although this has not been demonstrated experimentally; also, closely related strains of *R*. *felis* are neither fixed nor associated with sex-ratio distortion in their hosts, which are primarily cat fleas^45^. Parthenogenesis-induction is a common strategy found in maternally transmitted bacterial endosymbionts of insects, including *Wolbachia*^13^, *Cardinium*^14^ and *Rickettsia*^15^,^46^. This reproductive manipulation is an effective strategy for maternally transmitted symbionts as it increases the frequency of the transmitting sex (i.e. females). Two unrelated strains of *Rickettsia* have been shown to induce parthenogenesis in two eulophid wasps, *Neochrysocharis formosa*^15^ and *Pnigalio soemius*^46^. However, although many parthenogenetic insects are infected with endosymbionts, it is often difficult to demonstrate that these symbionts are the cause of the parthenogenesis, with the only exception being infections in haplodiploid insects^47^. This is because it is exceedingly difficult to establish new infections as these symbionts are intracellular and cannot be cultured. Also, removal of symbionts via antibiotic or heat treatment often does not restore sexual function. In the case of *L. bostrychophila*, it is difficult to remove *Rickettsia*, and treated booklice are typically sick and produce no offspring^11^,^12^. This has even led one group to suggest that *Rickettsia* might be an obligate symbiont of *L. bostrychophila*^11^. Therefore, the discovery of wild sexual *Rickettsia*-free *L. bostrychophila* promises to yield important insights into our understanding of the evolution and ecology of reproductive mode and symbiont infection in this important cosmopolitan pest species.

## Additional Information

**Accession codes**: DNA sequences: *12S* GenBank accession numbers: KF419246-KF419273, HM626248, HM626250, HM626255, KM454179-81. *16S* GenBank accession numbers: KF419223-KF419245, EU863798, EU863796, EU863792, FJ865400, GU563532, HM626262, HM626265, HM626271, HM626272, KM454182-3.

**How to cite this article**: Yang, Q. *et al.* Morphological and molecular characterization of a sexually reproducing colony of the booklouse *Liposcelis bostrychophila* (Psocodea: Liposcelididae) found in Arizona. *Sci. Rep.* **5**, 10429; doi: 10.1038/srep10429 (2015).

## Figures and Tables

**Table 1:** Morphological characters of parthenogenetic and sexual strains of *L. bostrychophila.*

***L. bostrychophila*Diagnostic characters**	♀ parthenogenetic^a^	♀ parthenogenetic Kansas strain n = 23 (OM), n = 34 (SEM)	♀ sexual Arizona strain n = 20 (OM), n = 21 (SEM)	♂ sexual Arizona strain n = 22 (OM), n = 22 (SEM)	*♂*sexual^b^ Hawaii strain
**Body length** (mm)	0.96-1.15	0.91-0.95	1.09-1.12	0.70-0.75	0.78-0.84
**Coloration**	Ochre to light brown, head sometimes slightly darker	Homogenously coloured, fallow to ochre, head slightly darker brown	Homogenously coloured chocolate to bistre, head slightly reddish-brown, middle part of anterior margin of tergits darker		Head reddish-brown, abdomen pale reddish brown, abdominal terga 3-7 with a slender transverse band of medium brown along anterior margin
**Maxillary palpus** (P4):	Sensillum s and r - long and slender thin-walled seta				Sensilla r and s long and setiform
**Eye:** - ommatidia numbers	(6)-7-(8)	7 ( = 2 oval, 5 round)	7 ( = 2 oval, 5 round)	5 ( = 2 oval, 3round)	5 ( = 2 oval, 3 round) * Eye:
**Eye:** - ommatidia surface structure	—	2 oval **- smooth** surface	**2 oval - granulated** surface	**2 oval - granulated** surface	2 oval **- smooth** surface
		5 round - smooth surface	5 round - smooth surface	3 round - smooth surface	3 round - smooth surface
**Vertex:**					
- tubercles	Medium to large size, usually smaller than alveoli of small fine hairs	Distinct; medium size, smaller than alveoli of small fine hairs	Very distinct; medium size, smaller than alveoli of small fine hairs		Distinct
- spindle-shaped areas	Usually well defined	Well defined, separated by lines of tubercles			Areoles of moderate width and distinct tubercles
- average hairs distance	Approximately 2x their length				Shorter than half the distance between their bases
**Lateral lobe of pronotum:**					
- SI	Short and pointed, not much longer than other small fine hairs of lateral lobe				Short, little longer than any other seta of pronotum
- other small hairs	3-7	(3)-4-**5**-6	(6)-**7**-8-9-(10)	3-4-**5**-6-(7)	—
**Prosternum:**					
- anterior half setae	3-4-(5)	(2)-3	3-4	**2**-3	2
- posterior half setae	2	2	2	2	2
**Mesosternum:** anterior setae	(6)-7-8-(9)	5-**6**-7	(6)-7-**8**-(9)	**5**-6	5
**Abdominal tergits**: 1 – 2	Each divided in 2 transverse bands				
**Abdominal tergits**: 3 – 7	Distinct tubercles of medium size, smaller than alveoli of small fine hairs. Each presenting a pale posterior membranous band with sculpture different from that on anterior portion of tergum				Each with anterior and posterior row of short setae on well sclerotized anterior region of tergum
**Abdominal tergits**: Average distance between short hairs	1.5-2x their length	1.5-3x their length			—
M10d and M10v	Differentiated				
SE	Differentiated				Differentiated, ~2x length of neighbouring setae
**Gonapophyses** (**♀**)	Common trunk - bifurcate			—	—
**Phallosome** (♂****): Basal rods	—	—	—	Not fused anteriorly (touched or almost touched)	Not fused, separated anteriorly (with interspace)

^a^based on the description by Günter (1974)^19^ and Lienhard (1990)^4^;

^b^based on the description by Mockford & Krushelnycky (2008)^16^; * supplementation according SEM (Kučerová).

OM=optical microscope; SEM=scanning electron microscope.

**Table 2:** Head width (W) measurements of the sexual Arizona strain (females, males) and an asexual Kansas strain (parthenogenetic females) of *Liposcelis bostrychophila.*

***Liposcelis bostrychophila***		**W (μm)**	
**mean ± SD**	**n**		
Sexual Arizona strain female	279.6 ± 7.3	15
Sexual Arizona strain male	202.1 ± 4.2	15
Asexual Kansas strain female	238.7 ± 9.2	19

Notes: W=the distance between the sides of the head measured behind the eyes, n=number of measured specimens.

**Table 3:** Samples used in the phylogenetic analysis.

**Reproduction type**	**Sex**	**Specimen Code**	**Locality**	**Collector / Reference**	**GenBank Nos.**	
					**16S rRNA**	**12S rRNA**
Sexual	Male	Lb_M1	Arizona, USA	Mockford, E.L.	KF419223	KF419248
		Lb_M2	Arizona, USA	Mockford, E.L.	KF419224	KF419249
		Lb_M3	Arizona, USA	Mockford, E.L.	KF419225	KF419250
		Lb_M4	Arizona, USA	Mockford, E.L.	KF419226	KF419251
		Lb_M5	Arizona, USA	Mockford, E.L.		KM454181
	Female	Lb_F1	Arizona, USA	Mockford, E.L.	KF419227	KF419252
		Lb_F2	Arizona, USA	Mockford, E.L.	KF419228	KF419253
		Lb_F3	Arizona, USA	Mockford, E.L.	KF419229	KF419254
		Lb_F4	Arizona, USA	Mockford, E.L.	KF419230	KF419255
		Lb_F5	Arizona, USA	Mockford, E.L.	KM454183	KM454180
		Lb_F6	Arizona, USA	Mockford, E.L.	KF419231	KF419256
		Lb_F7	Arizona, USA	Mockford, E.L.	KF419232	KF419257
		Lb_F8	Arizona, USA	Mockford, E.L.	KF419233	KF419258
		Lb_F9	Arizona, USA	Mockford, E.L.	KF419234	KF419259
		Lb_F10	Arizona, USA	Mockford, E.L.	KM454182	KM454179
Asexual (parthenogenesis)	Female	Lb_AU	Australia	Perlman,S.J.	HM626262	HM626248
		Lb_AZ	Tucson, Arizona, USA	Perlman, S.J.	HM626272	
		Lb_BB	Beibei, China	27	JN645276	JN645275
		Lb_BJ	Beijing, China	Cao, Y.	KF419235	KF419260
		Lb_BRE	Brisbane, Australia	Shao, R.	KF419236	KF419261
		Lb_CB	Central Bohemia, Czech	Kucerova, Z.	EU863798	KF419262
		Lb_DE	Germany	Adler, C.	KF419237	KF419263
		Lb_EB	Eastern Bohemia, Czech	Kucerova, Z.	KF419238	KF419264
		Lb_GX	Guangxi, China	Cao, Y.	EU863796	KF419265
		Lb_HR	Croatia	Kalinovic, I.	KF419239	KF419266
		Lb_IL.1	Illinois, USA	28	AY275368	AY275318
		Lb_IL.2	Illinois, USA	29	AY139944	AY139897
		Lb_IL.3	Illinois, USA	30	GU569226	
		Lb_KS.1	Kansas, USA	Opit, G.P.	GU563532	KF419267
		Lb_KS.2	Kansas, USA	Opit, G.P.	KF419240	KF419268
		Lb_MH	Manhattan, USA	Opit, G.P.	HM626265	HM626250
		Lb_SC	Seychelles	Liu, R.	KF419241	KF419269
		Lb_SY	Sanya, China	Li, Z.	KF419242	KF419270
		Lb_UK	United Kingdom	12		AJ428869
		Lb_VN	Vietnam	Gong, X.	KF419243	KF419271
		Lb_WP	Winnipeg	Perlman,S.J.	HM626271	HM626255
		Lb_XSG	Xinshaguang, China	Cao, Y.	KF419244	KF419272
		Lb_ZZ	Zhengzhou, China	Li, Z.	KF419245	KF419273
Sexual	—	Lc_CB	Central Bohemia, Czech	Kucerova, Z.	EU863792	KF419246
		Lc_KS	Kansas, USA	Opit, G.P.	FJ865400	KF419247

Lb refers to *Liposcelis bostrychophila* and Lc refers to *Liposcelis corrodens*.

**Table 4:** Primers used in this study.

**Target gene**	**Primer name**	**Sequence (5′-3′)**	**Reference**
Insect *12S*	12SF	TACTATGTTACGACTTAT	23
	12SR	AAACTAGGATTAGATACCC	
Insect *16S*	16Sar	CGCCTGTTTAACAAAAACAT	24
	16Sbr	CCGGTCTGAACTCAGATCACGT	
*Rickettsia 16S*	Rb-F	GCTCAGAACGAACGCTATC	31
	Rb-R	GAAGGAAAGCATCTCTGC	
